# Distributed Functional Connectome of White Matter in Patients With Functional Dyspepsia

**DOI:** 10.3389/fnhum.2021.589578

**Published:** 2021-04-16

**Authors:** Qiang Xu, Yifei Weng, Chang Liu, Lianli Qiu, Yulin Yang, Yifei Zhou, Fangyu Wang, Guangming Lu, Long Jiang Zhang, Rongfeng Qi

**Affiliations:** ^1^College of Automation Engineering, Nanjing University of Aeronautics and Astronautics, Nanjing, China; ^2^Department of Medical Imaging, Jinling Hospital, Medical School of Nanjing University, Nanjing, China; ^3^Department of Gastroenterology, Jinling Hospital, Medical School of Nanjing University, Nanjing, China; ^4^State Key Laboratory of Analytical Chemistry for Life Science, Nanjing University, Nanjing, China

**Keywords:** functional dyspepsia, functional connectome, white matter, resting-state fMRI, graph theory

## Abstract

**Purpose:** We aimed to find out the distributed functional connectome of white matter in patients with functional dyspepsia (FD).

**Methods:** 20 patients with FD and 24 age- and gender-matched healthy controls were included into the study. The functional connectome of white matter and graph theory were used to these participants. Two-sample *t*-test was used for the detection the abnormal graph properties in FD. Pearson correlation was used for the relationship between properties and the clinical and neuropshychological information.

**Results:** Patients with FD and healthy controls showed small-world properties in functional connectome of white matter. Compared with healthy controls, the FD group showed decreased global properties (Cp, S, Eglobal, and Elocal). Four pairs of fiber bundles that are connected to the frontal lobe, insula, and thalamus were affected in the FD group. Duration and Pittsburgh Sleep Quality Index positively correlated with the betweenness centrality of white matter regions of interest.

**Conclusion:** FD patients turned to a non-optimized functional organization of WM brain network. Frontal lobe, insula, and thalamus were key regions in brain information exchange of FD. It provided some novel imaging evidences for the mechanism of FD.

## Introduction

Functional dyspepsia (FD) is one of the most prevalent functional gastrointestinal disorders, with high prevalence (5–11% of the population) (Ford et al., 2015). FD is characterized by four main symptoms: bothersome postprandial fullness, early satiety, epigastric burning, and epigastralgia (Enck et al., 2017). FD negatively affects the quality of life in patients and is a healthcare burden for society for its recurrent nature of the symptoms (El-Serag and Talley, 2003; Lacy et al., 2011). In the absence of detectable organic causes, FD was referred to be a functional disorder, which was thought to result from the dysregulation in brain–gut interaction (Koloski et al., 2012). However, the neural basis of FD remains poorly understood.

Functional neuroimaging is a meaningful tool for identifying the human brain circuitry which correlates with the clinical phenotypic and behavioral manifestations in functional gastrointestinal disorder, including FD (Van Oudenhove et al., 2007; Lee et al., 2016; Kano et al., 2018). Based on the resting-state activity or task-based responses (e.g., visceral distention), the functional neuroimaging could assist in quantifying viscerosensory inputs that reach the brain. Several brain regions were reported in the research of neurological abnormalities in patients with FD, including somatosensory cortex, frontal cortex, insula, anterior cingulate cortex, thalamus, hippocampus, and amygdala (Al Omran and Aziz, 2014; Lee et al., 2016; Kano et al., 2018). Furthermore, a previous study suggested that people with gastric fundic distension showed altered frontal-limbic network (Ladabaum et al., 2007). Another study identified that the FD patients showed abnormal pain and salience network (Lee et al., 2016). These studies suggest that the brain network analysis could be a helpful tool for understanding the mechanism of brain alteration in patients with FD.

In general, the brain network could be derived from structural connectivity, functional connectivity, and effective connectivity among the distributed brain regions (Bullmore and Sporns, 2009). Functional connectivity mainly could be built by temporal correlation or coherences between signals from brain regions (Achard and Bullmore, 2007; Biswal et al., 2010). Functional brain network analysis has been applied to the research of cognition and brain diseases/disorders (He and Evans, 2010; Ding et al., 2011; Zhang et al., 2011; Gao et al., 2013). However, most of the previous studies evaluated the functional connectivity of gray matter in blood oxygen level–dependent (BOLD) fMRI. The information of white matter (WM) in BOLD-fMRI has been ignored because the signals in white matter were thought to be noisy, unreliable, and undetectable in historical period. The role of WM in neuroimaging is still controversial.

Recently, several studies have detected the brain activity in WM in BOLD-fMRI. Evidences from amplitude and connectivity studies demonstrated that the signal of WM in BOLD-fMRI exhibited a specific distribution rather than a random distribution of noise: the WM functional connectomes exhibited a reliable and stable small-world topology, and the abnormal amplitude of low-frequency fluctuation (ALFF) in WM could provide the evidence for understanding the functional role of fiber tracts in the pathology of Parkinson’s disease (Ji et al., 2017; Li et al., 2019). Moreover, WM activity was shown to be modulated under different cognitive tasks (Ji et al., 2017; Wu et al., 2017; Huang et al., 2018). In Alzheimer’s disease, the WM function was associated with the regional glucose metabolism and correlated with memory function (Makedonov et al., 2016). Another study found that patients with Parkinson’s disease showed increased small-worldness in the functional network of WM (Ji et al., 2019). It suggested that the functional connectivity of WM could be a useful and novel tool for investigating the alteration in brain disorders. In this study, we aimed to reveal the functional connectome of WM in FD patients. It might be helpful for the mechanism investigation with a novel insight.

## Materials and Methods

### Participants

Twenty patients with functional dyspepsia (FD) (14 female, age range: 20–62 years, 40.80 ± 12.22 years) and 24 healthy controls (16 female, age range: 21–68 years, 42.29 ± 15.66 years) were included in this study. All participants were right-handed and provided written informed consent before the whole study began. The study was approved by the Medical Research Ethics Committee of Jinling Hospital in accordance with the Helsinki Declaration (Approval no. 2016NZGKJ-070).

The FD patients were diagnosed by the gastroenterologist from the Digestive Disease Clinic of Jinling Hospital by following the Rome III criteria (Drossman, 2006). The gastroenterologist has extensive experience in functional gastrointestinal disorders. The exclusion criteria were as follows: a history of gastrointestinal surgery; major medical or neurological conditions; psychiatric disorders or substance abuse; any previous treatment with centrally acting medications such as aspirin and selective serotonin reuptake inhibitors. All patients were assessed with the Pittsburgh Sleep Quality Index (PSQI). The healthy controls were recruited from the local community through printed advertisements.

### MRI Data Acquisition

MRI data were collected by using a 3T MR scanner (Tim Trio, Siemens, Germany). The participants were instructed to stay still during scanning and keep their eyes closed but not fall asleep. Resting-state BOLD fMRI and high-resolution T1-weighted structural image were scanned during the study. The parameters of fMRI were as follows: TR/TE = 2,000/30 ms; FOV = 240 mm × 240 mm; matrix = 64 × 64; thickness = 4 mm with a gap of 0.4 mm between slices, 30 axial slices, with 250 brain volumes (500 s). The parameters of structural images were as follows: TR/TE = 2,300/2.98 ms; field of view = 256 mm × 256 mm; matrix size = 256 × 256, 176 sagittal slices with thickness of 1 mm, no gap between slices.

### Data Preprocess

Functional images were preprocessed by using DPARSF (v4.3^1^) (Chao-Gan and Yu-Feng, 2010) and SPM12 toolkit^2^. After excluding 10 volumes, slice-timing correction and realignment were applied to the 240 left functional volumes. Subjects were excluded if his or her head motion exceeded 2.0 mm translation or 2.0° rotation. The mean frame-wise displacement (FD) was also calculated for each subject. Individuals with head motion of > 1.0 mm in translation and 1.0° in rotation were excluded. None of the participants was excluded for the head motion.

Structural images were then co-registered with the preprocessed functional images (mean functional image for each subject) and segmented into gray matter (GM), WM, and cerebrospinal fluid (CSF) using a diffeomorphic non-linear registration algorithm (DARTEL) (Ashburner, 2007) in SPM12. The mean CSF signals from 95% threshold cut-off mask, 24 head motion parameters (6 head motion parameters, 1 time point before, and the 12 corresponding squared items), and scrubbing parameters (FD > 0.5 mm along with one-forward and two-back neighbors) were regressed out from functional data. To avoid elimination of important neural signals, we did not remove or regress out WM or global signals (Ji et al., 2017; Li et al., 2019). To minimize mixing signal (and noise) components from the WM regions due to partial volume effect, subsequent processing of the functional images was performed for WM in accordance with previous study parameters (Li et al., 2019). First, the individual masks were generated using a rigorous 90% threshold on the probability map of WM. Functional images were spatially separated into WM images using the dot product between functional images and individual WM mask. Then the functional images in WM were spatially normalized onto Montreal Neurological Institute (MNI) space using DARTEL normalization operation and resampled to 3 mm × 3 mm × 3 mm. To minimize spurious local spatial correlations between voxels, spatial smoothing was not applied. Subsequently, linear trending and band-pass filtering (0.01–0.10 Hz) were performed to minimize any drifts as well as minimize high-frequency physiological noise sources such as the respiration rate.

Next, the individual WM masks were spatially normalized onto MNI space using DARTEL normalization operation and resampled to 3 mm × 3 mm × 3 mm. Then, only voxels identified as WM across 80% of subjects were included as part of the group-level WM mask (Li et al., 2019). To exclude the impact of deep brain structures, the probability (25% threshold) Harvard–Oxford Atlas was used to remove subcortical nuclei (i.e., bilateral thalamus, putamen, caudate, pallidum, and nucleus accumbens) from the group-level WM mask.

### Parcellation of WM

The group-level WM mask was subdivided into 128 random regions of interest (ROIs) (Ji et al., 2019; Li et al., 2019) and was generated and approximately identical in size (mean ± SD = 99.24 ± 0.43 voxels across ROIs), as previously described by Zalesky et al. (2010). The WM group parcellation used here was attached as Supplementary Information Table 1 and marked using JHU-Atlas.

### Functional Connectome of WM

**FIGURE 1:**
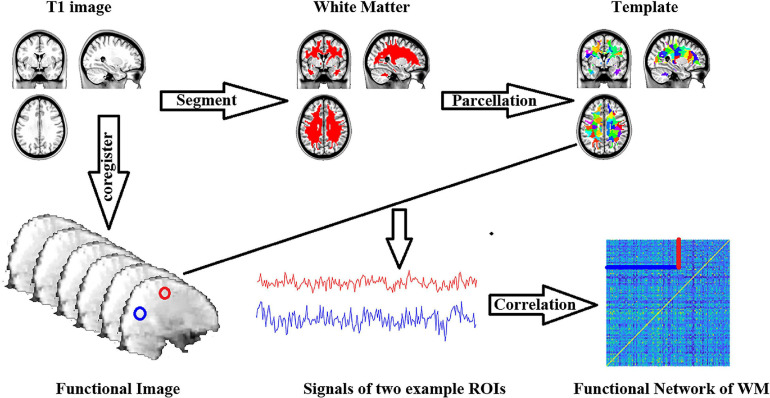
Workflow of functional connectome of white matter construction.

Pearson’s correlation coefficient was used between each ROI’s averaged time series. Fisher’s r to Z transformation was applied to each of the correlation matrices. A schematic of the analyses is shown in Figure 1. Finally, we estimated the topological properties of the WM functional connectome.

### Network Properties of Functional Connectome of WM

#### Threshold Selection

To explore the influence of thresholds on topological properties (Bullmore and Bassett, 2011), we used sparsity-based (proportional) thresholds to the weighted correlation matrix corresponding to each subject (Garrison et al., 2015). The sparsity was defined as the ratio of the real edge numbers divided by the maximum possible edge numbers in a given network at r_*thr*_. We decreased the r_*thr*_ from 1 to 0 (from maximum to minimum) until the existing number of edgessatisfied a sparsity threshold. Specifically,

$$0\leq \mathrm{sparsity}\leq 1=\frac{\varepsilon_{\mathrm{thr}}}{N\,\left(N-1\right)/2}$$

Where ε_*thr*_expressed the existing number of edges generated by threshold at r_*thr*_, and N(N − 1)/2 represents the maximum possible number of edges existing in a given network of N nodes (Bullmore and Bassett, 2011; Liao et al., 2018). In this case, when r_*thr*_ = 0, sparsity = 1; when r_*thr*_ = 1, sparsity = 0.

#### Topological Properties of WM Functional Network

The global and nodal topological properties of WM functional connectome were computed using Gretna software (v2.0^3^). The following global parameters were included: strength of network (S), global efficiency (Eglobal), local efficiency (Elocal), clustering coefficient (Cp), the shortest path length (Lp), normalized clustering coefficient γ()normalized shortest path length λ()and small-worldness σ()Here, S measured the connectivity capacity of the network, Eglobal quantified the capacity of information exchange across the whole network, and Elocal was the measurement of the fault tolerance of the subgraph, showing the efficiency of information exchange at the local level. The small-worldness supported both segregated and intergrated information processing.

Meanwhile, the following nodal parameters were included in the study: betweenness centrality (BC), strength (Snodal), and efficiency (Enodal). BC represents the node ability of bridging the disparate parts of the network. Snodal measures the connectivity capacity of the node, and Enodal measures the capacity of information exchange of the node. A review outlined the uses and interpretations of these topological properties (Rubinov and Sporns, 2010). The definitions of these properties are described in the Supplementary File.

### Statistical Analysis

The statistical analysis of the demographic and neuropsychological data was carried on by using GraphPad Prism^4^. The differences of age between two groups were tested by two-sample *t*-test. Also, the sex difference was tested by χ^2^ test.

The statistical analysis of the global properties was carried by using SurfStat toolbox^5^. The differences of each sparsity and those of the AUC (area under curve) were test by two-sample *t*-test under the model of general linear model. The differences of the nodal properties were tested only on the AUC condition by using the same method of the global properties.

The Pearson correlation analysis was used to find the relationship between the clinical information, neuropsychological table, and network properties.

## Results

### Demographic and Neuropsychological Data

There was no significant difference in age and sex between the FD patients and healthy controls (Table 1).

**TABLE 1:** Demographic and neuropsychological data.

	**FD**	**HC**	**Statistical results**
Sex	14F/6M	16F/8M	χ^2^ = 0.056, *p* = 0.813
Age (years)	40.80 ± 12.22	42.29 ± 15.66	*t* = −0.347, *p* = 0.731
Duration (months)	42.03 ± 75.92	–	–
PSQI	8.15 ± 4.32	–	–

### Group Differences of Global Properties

**FIGURE 2:**
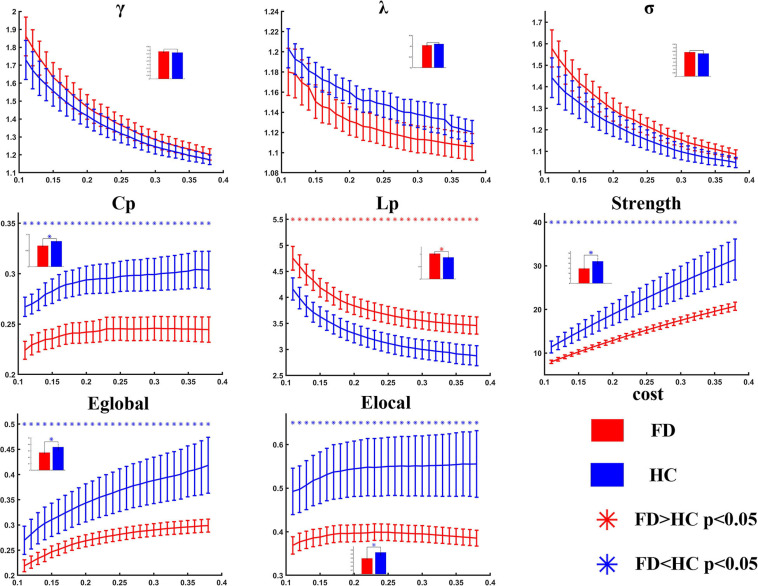
Statistical analysis for the global properties between functional dyspepsia (FD) and healthy controls (HC). Compared with HC, the FD patients showed decreased Cp, strength, Eglobal, and Elocal, and increased Lp.

Compared with the healthy controls, the FD patients showed significant decreases in Cp (*t* = −3.303, *p* = 0.001 in AUC model), S (*t* = −2.184, *p* = 0.017 in AUC model), Eglobal (*t* = −1.969, *p* = 0.028 in AUC model), and Elocal (*t* = −2.116, *p* = 0.020 in AUC model), and showed a significant increase in Lp (*t* = 2.595, *p* = 0.007 in AUC model). The differences could be detected on both AUC mode and sparsity mode. No significant difference was found in γ, λ, and σ either on AUC or on sparsity (Table 2 and Figure 2).

**TABLE 2:** Group differences of small-world property.

	**γ**	**λ**	**σ**	**Cp**	**Lp**	**Strength**	**Eglobal**	**Elocal**
T values	–1.013	0.773	1.078	–3.303	2.595	–2.184	–1.969	–2.116
*P* values	0.159	0.222	0.144	0.001*	0.007*	0.017*	0.028*	0.020*

*Significant alterations.

### Group Differences of Nodal Properties

**FIGURE 3:**
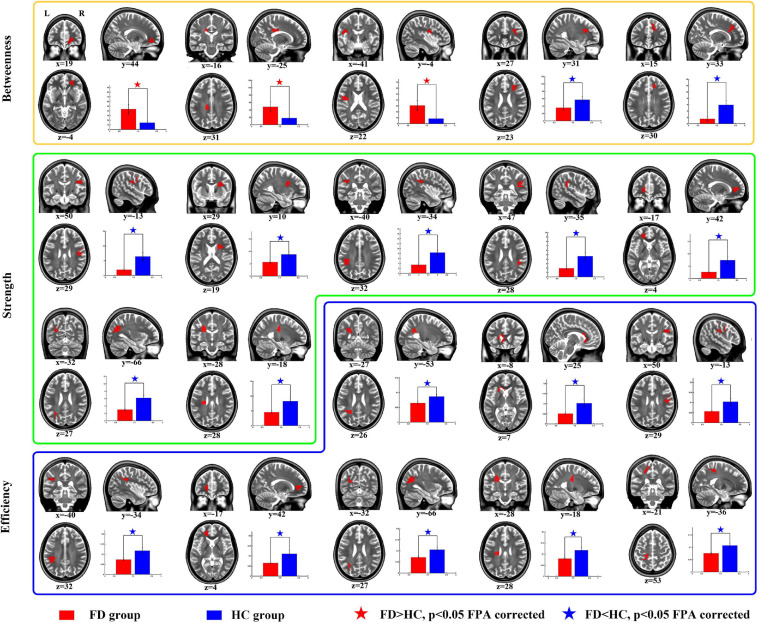
Group differences of nodal properties between FD and HC. Compared with HC, the FD patients showed alteration in anterior corona radiata, corpus callosum, superior longitudinal fasciculus, and posterior thalamic radiation.

For the comparison of nodal BC, the FD patients showed increases on nodes 2 (located on the right anterior corona radiata), 13 (located on the body of corpus callosum), and 42 (located on the left superior longitudinal fasciculus), and showed decreases on nodes 57 (located on the right anterior corona radiata) and 110 (located on genu of corpus callosum) (Table 3 and Figure 3).

**TABLE 3:** Group differences of nodal property.

**Nodal order**	**MNI coordinates**			**Localization in JHU-Atlas**	**T**	**P**
	**x**	**y**	**z**			
**Betweenness**						
2	21	45	−6	Anterior_corona_radiata_R	2.847	0.003
13	−15	−30	30	Body_of_corpus_callosum	2.837	0.004
42	−42	−6	21	Superior_longitudinal_fasciculus_L	2.730	0.005
57	30	27	21	Anterior_corona_radiata_R	–2.818	0.004
110	15	33	33	Genu_of_corpus_callosum	–2.722	0.005
**Strength**						
12	48	−12	30	Superior_longitudinal_fasciculus_R	–2.653	0.006
15	27	9	21	Anterior_corona_radiata_R	–2.534	0.008
18	−42	−39	33	Superior_longitudinal_fasciculus_L	–2.915	0.003
27	45	−39	27	Superior_longitudinal_fasciculus_R	–2.699	0.005
64	−18	45	3	Anterior_corona_radiata_L	–2.871	0.003
84	−33	−63	27	Posterior_thalamic_radiation_(include_optic_radiation)_L	–3.432	0.001
99	−27	−18	30	Superior_corona_radiata_L	–2.944	0.003
**Efficiency**						
8	−27	−51	24	Posterior_corona_radiata_L	–3.003	0.002
9	−12	24	9	Genu_of_corpus_callosum	–2.554	0.007
12	48	−12	30	Superior_longitudinal_fasciculus_R	–2.536	0.008
18	−42	−39	33	Superior_longitudinal_fasciculus_L	–2.604	0.006
64	−18	45	3	Anterior_corona_radiata_L	–2.729	0.005
84	−33	−63	27	Posterior_thalamic_radiation_(include_optic_radiation)_L	–2.544	0.007
99	−27	−18	30	Superior_corona_radiata_L	–2.952	0.003
106	−21	−36	54	Unclassified	–2.831	0.004

For the comparison of nodal strength, the FD patients showed decreases on nodes 12 (located on the right superior longitudinal fasciculus), 15 (located on the right anterior corona radiata), 18 (located on the left superior longitudinal fasciculus), 27 (located on the right superior longitudinal fasciculus), 64 (located on the left anterior corona radiata), 84 (located on the left posterior thalamic radiation), and 99 (located on left superior corona radiata) (Table 3 and Figure 3).

For the comparison of nodal efficiency, the FD patients showed decreases on nodes 8 (located on the left posterior corona radiata), 9 (located on genu of corpus callosum), 12 (located on the right superior longitudinal fasciculus), 18 (located on the left superior longitudinal fasciculus), (located on the left anterior corona radiata), 84 (located on the left posterior thalamic radiation), 99 (located on left superior corona radiata), and 106 (unclassified on the JHU-Atlas, but located near left posterior corona radiata) (Table 3 and Figure 3). All results of node comparison were corrected by false-positive adjustment (Fornito et al., 2011; Jao et al., 2013; Jin et al., 2014).

### Correlation Between Clinical Information, Neuropsychological Table, and Network Properties

**FIGURE 4:**
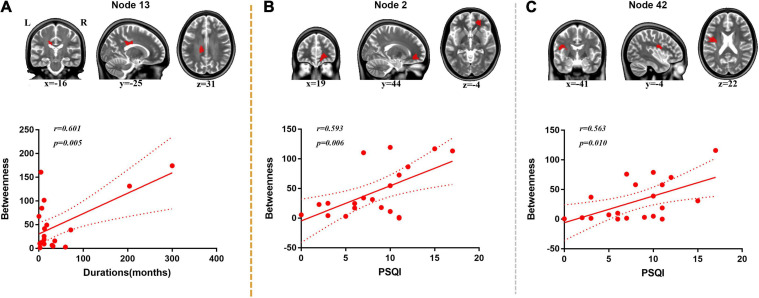
Correlation analysis between duration, PSQI, and nodal BC. **(A)** Positive correlation was found between duration and BC of node 13 (body of corpus callosum). **(B)** Positive correlation was found between PSQI and BC of node 2 (right anterior corona radiata). **(C)** Positive correlation was found between PSQI and BC of node 42 (left superior longitudinal fasciculus).

A positive correlation was found between duration of disorder and BC value of node 13 (located on the body of corpus callosum, *r* = 0.601, *p* = 0.005) (Figure 4).

Positive correlations were found between PSQI and BC values of node 2 (located on the right anterior corona radiata, *r* = 0.593, *p* = 0.006) and node 42 (located on the left superior longitudinal fasciculus, *r* = 0.563, *p* = 0.010) (Figure 4).

## Discussion

In this study, we applied the functional connectome of WM to discover the alteration of patients with FD. Both FD and HC groups showed small-world properties in the functional connectome of WM. Compared with the HC group, the FD patients showed significant decreases in global properties (Cp, S, Eglobal, and Elocal). Moreover, four pairs of fibers were affected in FD patients in the nodal properties comparison. The duration and PSQI also correlated with the alteration of nodal properties.

Graph theory provided a network prospective to investigate how the brain works interactively. The human brain is organized in a “small-world” pattern with high value and low energetic cost (Bullmore and Sporns, 2009). Small-world properties have been used to detect the alteration in brain disease, such as Alzheimer’s disease (Phillips et al., 2015; Vecchio et al., 2018), epilepsy (Zhang et al., 2011; Ji et al., 2015), and stroke (Case et al., 2019). In this study, we investigated the functional architecture of WM using resting-state fMRI in FD patients. It fitted the former studies that the functional connectome of WM had a small-world structure (Ji et al., 2019; Li et al., 2019). In both patients and healthy controls, there was a small-world property in the WM functional network. As we know, this was the first time that the graph theory was applied to analyze the WM functional network in FD researches. In our result, the FD patients showed decreased strength, efficiency, and clustering coefficient, which implied that the FD patients showed a non-optimized structure of WM network in both global and local level properties. These findings were partially consistent with our former study in irritable bowel syndrome (IBS) patients where they showed decreased global efficiency compared with healthy controls (Qi et al., 2016a). Also, the decreased efficiency was found in other chronic pain, such as postherpetic neuralgia (Zhang et al., 2014). It suggested that the functional gastrointestinal disorders may show the information process efficiency loss.

In nodal-level statistical analysis, there were alterations in four pairs of fiber bundles in patients with FD: the anterior corona radiata, corpus callosum, superior longitudinal fasciculus, and posterior thalamic radiation. It was consistent with the findings of WM in FD patients in a former DTI study, which showed alteration in corona radiata, internal capsule, posterior thalamic radiation, corpus callosum, external capsule, sagittal stratum, and superior longitudinal fasciculus (Zhou et al., 2013). A similar alteration in WM microstructure could be found in other kinds of chronic pain, such as IBS (Chen et al., 2011), migraine (Szabó et al., 2012), and temporomandibular disorder (Moayedi et al., 2012). Besides, the anterior corona radiata was the connecting fiber bundle within the frontal lobe, the superior longitudinal fasciculus was the connecting fiber bundle from frontal lobe to insula and ends in the posterior part of the brain, the posterior thalamic radiation was the ascending fiber bundle from thalamus to cerebral cortex, and the corpus callosum was the connecting fiber bundle to bridge two hemispheres. These results also fitted the functional alteration in GM-related studies, such as decreased functional connectivity of insula (Sun et al., 2020); decreased GM density in the middle frontal gyrus, right precentral gyrus, and insula (Zeng et al., 2013); and decreased connectivity between insula and thalamus (Liu et al., 2018). Our former study also found altered amplitude of low-frequency fluctuation in insula and thalamus (Qi et al., 2020). Our results supported that the frontal lobe, insula, and thalamus were the key regions in FD patients.

In addition, correlation analysis found that the nodal BC was correlated with the duration and PSQI. No significant correlation was found between nodal properties S and efficiency. BC represents the ability of bridging different parts of the brain network (Rubinov and Sporns, 2010). The increased betweenness centrality of corpus callosum represents that the information exchange between two hemispheres was enhanced, and the positive correlation with the duration demonstrates that the duration affected this process. It was consistent with our former study of interhemispheric functional connectivity in IBS (Qi et al., 2016b). The two enhanced betweenness centrality nodes were located in the anterior corona radiata and superior longitudinal fasciculus, which were near the frontal lobe and insula (Zeng et al., 2013; Liu et al., 2018; Sun et al., 2020). The positive correlation with PSQI represents that the frontal lobe and insula might be important in the modulation of sleep quality in FD patients.

Different with the GM connectome and WM structural network, the alterations of connectome of WM might provide novel and more information for description of the mechanism of FD. Compared with the GM connectome, the WM functional network showed a tendency toward randomization (Li et al., 2019). Previous studies showed that the effect of deoxygenated blood drainage from GM, through WM to the deep venous system, was less than 3% (Ruíz et al., 2009; Huang et al., 2018). The WM functional connectome showed more relationship to the WM structural network. According to the previous studies, the ALFF of WM showed significant correlation with FA in healthy subjects (Ji et al., 2017), but the alterations of ALFF of WM and FA were different in Parkinson’s disease (Ji et al., 2019). However, the WM connectome was the promising neuromarker for the brain–behavior prediction (Li et al., 2020a). Also, it was the potential neuromarker for the classification of psychological disorder (Li et al., 2020b). It suggested that the WM connectome could extend the width of neuroimaging insight for understanding the pathophysiological mechanism for the disease.

## Limitation

This study has several limitations. First, our results were based on a relatively small sample size and therefore should be considered preliminary. Further studies should contain more participants and even could be separated into different subtypes. Second, in this preliminary study, we did not have the diffusion MRI for further support. Third, additional results of graph theory analysis were not listed here. Further studies would consider the combined connectivity of GM and WM.

## Conclusion

In this study, we found that the functional connectome of WM in FD patients turned to a non-optimized regularity in both global and local level. Also, the abnormal nodes were mainly located near the frontal lobe, insula, and thalamus. These findings provided a new prospective for the mechanism of FD.

## Data Availability Statement

The raw data supporting the conclusions of this article will be made available by the authors, without undue reservation.

## Ethics Statement

The studies involving human participants were reviewed and approved by the Medical Research Ethics Committee of Jinling hospital. The patients/participants provided their written informed consent to participate in this study.

## Author Contributions

QX and YW were involved in literature review, experimental design, data analysis, and writing of the manuscript. CL contributed to the data collection and the analysis of neuropsychological data. YW, LQ, and CL contributed to the data collection. FW contributed in the experimental design. LZ and RQ contributed in the experimental design and revision of the manuscript. GL was involved in the experimental design and revision of the manuscript. All authors contributed to the article and approved the submitted version.

## Conflict of Interest

The authors declare that the research was conducted in the absence of any commercial or financial relationships that could be construed as a potential conflict of interest.

## Footnotes

1 https://www.restfmri.net/

2 https://www.fil.ion.ucl.ac.uk/spm/

3 https://www.nitrc.org/projects/Gretna

4 http://www.graphpad.com

5 http://www.math.mcgill.ca/keith/surfstat/#ICBMagain

## Supplementary Material

Supplementary Table 1 Node position and localization in JHU-Atlas.
