# Supplementary material for: Distributed Functional Connectome of White Matter in Patients With Functional Dyspepsia
Source: Front Hum Neurosci. 2021 Apr 16;15:589578. doi: 10.3389/fnhum.2021.589578 (PMC8085333; doi:10.3389/fnhum.2021.589578)
Supplement: Supplementary Table 1 — Node position and localization in JHU-Atlas. [file Table_1.DOCX]

Supplemental Table S1

Table S1 Node position and localization in JHU-atlas

| Nodal Order | MNI Coordinate | | | JHU-Atlas |
| --- | --- | --- | --- | --- |
|  | x | y | z |  |
| 1 | -45 | -33 | -15 | Sagittal_stratum_(include_inferior_longitidinal_  fasciculus_and_inferior_fronto-occipital_fasciculus)_L |
| 2 | 21 | 45 | -6 | Anterior_corona_radiata_R |
| 3 | -33 | -57 | 0 | Posterior_thalamic_radiation_(include_optic_radiation)_L |
| 4 | 0 | -24 | 27 | Body_of_corpus_callosum |
| 5 | -21 | 33 | 0 | Anterior_corona_radiata_L |
| 6 | -18 | -63 | -33 | Middle_cerebellar_peduncle |
| 7 | 15 | 42 | 24 | Genu_of_corpus_callosum |
| 8 | -27 | -51 | 24 | Posterior_corona_radiata_L |
| 9 | -12 | 24 | 9 | Genu_of_corpus_callosum |
| 10 | -15 | -21 | 63 | Unclassified |
| 11 | -24 | -24 | 51 | Unclassified |
| 12 | 48 | -12 | 30 | Superior_longitudinal_fasciculus_R |
| 13 | -15 | -30 | 30 | Body_of_corpus_callosum |
| 14 | -36 | -21 | 24 | External_capsule_L |
| 15 | 27 | 9 | 21 | Anterior_corona_radiata_R |
| 16 | 33 | 36 | 3 | Anterior_corona_radiata_R |
| 17 | -18 | 21 | 27 | Anterior_corona_radiata_L |
| 18 | -42 | -39 | 33 | Superior_longitudinal_fasciculus_L |
| 19 | 42 | 3 | -30 | Anterior_corona_radiata_R |
| 20 | -9 | 30 | 9 | Genu_of_corpus_callosum |
| 21 | 36 | -3 | 18 | External_capsule_R |
| 22 | -18 | -54 | 30 | Posterior_corona_radiata_L |
| 23 | -39 | -6 | 39 | Superior_longitudinal_fasciculus_L |
| 24 | 30 | -33 | 12 | Retrolenticular_part_of_internal_capsule_R |
| 25 | 39 | -42 | 33 | Superior_longitudinal_fasciculus_R |
| 26 | -21 | -9 | 33 | Superior_corona_radiata_L |
| 27 | 45 | -39 | 27 | Superior_longitudinal_fasciculus_R |
| 28 | 21 | -57 | -39 | Middle_cerebellar_peduncle |
| 29 | 18 | -3 | 48 | Superior_corona_radiata_R |
| 30 | -12 | -39 | 24 | Splenium_of_corpus_callosum |
| 31 | 54 | -18 | 3 | Posterior_limb_of_internal_capsule_R |
| 32 | -36 | -6 | -27 | Cerebral_peduncle_L |
| 33 | 24 | -69 | 24 | Posterior_thalamic_radiation_(include_optic_radiation)_R |
| 34 | -27 | 21 | 18 | Anterior_corona_radiata_L |
| 35 | -24 | -57 | -39 | Middle_cerebellar_peduncle |
| 36 | 24 | -24 | -18 | Splenium_of_corpus_callosum |
| 37 | 42 | 39 | -6 | Anterior_corona_radiata_R |
| 38 | -12 | -9 | 54 | Body_of_corpus_callosum |
| 39 | 30 | -18 | 54 | Superior_corona_radiata_R |
| 40 | -42 | 0 | -27 | External_capsule_L |
| 41 | -27 | -54 | 15 | Posterior_thalamic_radiation_(include_optic_radiation)_L |
| 42 | -42 | -6 | 21 | Superior_longitudinal_fasciculus_L |
| 43 | 30 | -9 | -3 | Anterior_limb_of_internal_capsule_R |
| 44 | -33 | -27 | 42 | Superior_longitudinal_fasciculus_L |
| 45 | -30 | -12 | 9 | Superior_longitudinal_fasciculus_L |
| 46 | -39 | -54 | 12 | Superior_longitudinal_fasciculus_L |
| 47 | 39 | 0 | 27 | Superior_longitudinal_fasciculus_R |
| 48 | 18 | 30 | -6 | Anterior_corona_radiata_R |
| 49 | -45 | -42 | 0 | Superior_longitudinal_fasciculus_L |
| 50 | -15 | 51 | -9 | Unclassified |
| 51 | -6 | -3 | 30 | Cingulum_(cingulate_gyrus)_L |
| 52 | 21 | -51 | 45 | Posterior_corona_radiata_R |
| 53 | 27 | 0 | 39 | Superior_corona_radiata_R |
| 54 | 12 | -9 | 33 | Body_of_corpus_callosum |
| 55 | -12 | -51 | -33 | Middle_cerebellar_peduncle |
| 56 | 18 | -42 | 24 | Splenium_of_corpus_callosum |
| 57 | 30 | 27 | 21 | Anterior_corona_radiata_R |
| 58 | 48 | -36 | -12 | Sagittal_stratum_(include_inferior_longitidinal_  fasciculus_and_inferior_fronto-occipital_fasciculus)_R |
| 59 | 9 | -6 | -6 | Body_of_corpus_callosum |
| 60 | -24 | 12 | 18 | Anterior_limb_of_internal_capsule_L |
| 61 | -15 | -69 | -33 | Posterior_thalamic_radiation_(include_optic_radiation)_L |
| 62 | 27 | -33 | 48 | Unclassified |
| 63 | -33 | 36 | 3 | Anterior_corona_radiata_L |
| 64 | -18 | 45 | 3 | Anterior_corona_radiata_L |
| 65 | 9 | -51 | -24 | Splenium_of_corpus_callosum |
| 66 | 39 | -39 | -3 | Retrolenticular_part_of_internal_capsule_R |
| 67 | -15 | 9 | 33 | Superior_corona_radiata_L |
| 68 | 39 | -54 | -3 | Posterior_thalamic_radiation_(include_optic_radiation)_R |
| 69 | -21 | 33 | -9 | Anterior_corona_radiata_L |
| 70 | 36 | -54 | 27 | Superior_longitudinal_fasciculus_R |
| 71 | 51 | -27 | -18 | Retrolenticular_part_of_internal_capsule_R |
| 72 | 33 | -33 | 24 | Posterior_corona_radiata_R |
| 73 | 39 | -21 | 33 | Superior_longitudinal_fasciculus_R |
| 74 | -21 | -6 | 21 | Superior_corona_radiata_L |
| 75 | 21 | -36 | 39 | Posterior_corona_radiata_R |
| 76 | -15 | -54 | 45 | Unclassified |
| 77 | -33 | 51 | -6 | Anterior_corona_radiata_L |
| 78 | -42 | -18 | 30 | Superior_longitudinal_fasciculus_L |
| 79 | 27 | -75 | 15 | Posterior_thalamic_radiation_(include_optic_radiation)_R |
| 80 | 15 | 15 | 42 | Body_of_corpus_callosum |
| 81 | -18 | 33 | 30 | Anterior_corona_radiata_L |
| 82 | -21 | 3 | 39 | Superior_corona_radiata_L |
| 83 | 24 | -12 | 36 | Superior_corona_radiata_R |
| 84 | -33 | -63 | 27 | Posterior_thalamic_radiation_(include_optic_radiation)_L |
| 85 | 24 | 45 | 9 | Anterior_corona_radiata_R |
| 86 | 15 | -39 | 54 | Unclassified |
| 87 | -48 | -24 | -6 | Retrolenticular_part_of_internal_capsule_L |
| 88 | -15 | 0 | 48 | Superior_corona_radiata_L |
| 89 | 30 | -72 | 0 | Posterior_thalamic_radiation_(include_optic_radiation)_R |
| 90 | 15 | 36 | 3 | Anterior_corona_radiata_R |
| 91 | -6 | -12 | 36 | Cingulum_(cingulate_gyrus)_L |
| 92 | 42 | -21 | -12 | Sagittal_stratum_(include_inferior_longitidinal_  fasciculus_and_inferior_fronto-occipital_fasciculus)_R |
| 93 | 9 | 24 | 0 | Genu_of_corpus_callosum |
| 94 | 27 | -15 | 45 | Superior_corona_radiata_R |
| 95 | -18 | -45 | -39 | Middle_cerebellar_peduncle |
| 96 | 9 | -33 | 27 | Body_of_corpus_callosum |
| 97 | 48 | -9 | -24 | Anterior_corona_radiata_R |
| 98 | 21 | -48 | -36 | Middle_cerebellar_peduncle |
| 99 | -27 | -18 | 30 | Superior_corona_radiata_L |
| 100 | -18 | -33 | 45 | Superior_corona_radiata_L |
| 101 | -15 | -39 | -33 | Cerebral_peduncle_L |
| 102 | -33 | -33 | 6 | Retrolenticular_part_of_internal_capsule_L |
| 103 | 24 | -57 | 15 | Splenium_of_corpus_callosum |
| 104 | 15 | -84 | 0 | Posterior_thalamic_radiation_(include_optic_radiation)_L |
| 105 | 36 | -48 | 3 | Posterior_thalamic_radiation_(include_optic_radiation)_R |
| 106 | -21 | -36 | 54 | Unclassified |
| 107 | 12 | -63 | -33 | Superior_cerebellar_peduncle_R |
| 108 | -9 | 39 | -18 | Unclassified |
| 109 | -36 | -42 | 27 | Superior_longitudinal_fasciculus_L |
| 110 | 15 | 33 | 33 | Genu_of_corpus_callosum |
| 111 | 24 | 21 | 36 | Anterior_corona_radiata_R |
| 112 | -48 | -9 | -15 | External_capsule_L |
| 113 | 30 | -9 | 27 | Superior_corona_radiata_R |
| 114 | 24 | 30 | 15 | Anterior_corona_radiata_R |
| 115 | 18 | 6 | 30 | Body_of_corpus_callosum |
| 116 | -21 | -84 | 6 | Posterior_thalamic_radiation_(include_optic_radiation)_L |
| 117 | 15 | 18 | 30 | Body_of_corpus_callosum |
| 118 | -27 | -30 | 15 | Retrolenticular_part_of_internal_capsule_L |
| 119 | 36 | -3 | -27 | Cerebral_peduncle_R |
| 120 | -18 | 42 | 24 | Anterior_corona_radiata_L |
| 121 | -15 | -39 | 15 | Posterior_corona_radiata_L |
| 122 | 27 | -54 | 24 | Posterior_corona_radiata_R |
| 123 | -36 | -21 | -6 | Retrolenticular_part_of_internal_capsule_L |
| 124 | 24 | -15 | 15 | Posterior_limb_of_internal_capsule_R |
| 125 | 15 | -78 | 9 | Splenium_of_corpus_callosum |
| 126 | -30 | 9 | 36 | Superior_corona_radiata_L |
| 127 | 27 | -30 | 36 | Posterior_corona_radiata_R |
| 128 | -18 | -60 | 36 | Posterior_corona_radiata_L |
