# Supplementary material for: Distributed Functional Connectome of White Matter in Patients With Functional Dyspepsia
Source: Front Hum Neurosci. 2021 Apr 16;15:589578. doi: 10.3389/fnhum.2021.589578 (PMC8085333; doi:10.3389/fnhum.2021.589578)
Supplement: Supplementary file 2 [file Table_2.docx]

Supplemental information

Definitions of the network properties

Clustering coefficient of a node i was defined as the extent to which the neighborhoods were connected with each other or not, and was computed as follows:

$$C\left( i \right)=\frac{\sum_{j,h\in N} \left( w_{ij}w_{ih}w_{jh} \right)^{1/3}}{k_{i}(k_{i}-1)}$$

Where $k_{i}$ is the number of nodes connected to node i, and w is the weight scaled by the mean of all weights to control each participant’s cost at the same level. The Cp of a network is the average clustering coefficient over all nodes, and indicates the extent of the local inter-connectivity or cliquishness in a network.

The path length between any pair of nodes (e.g., i and j) was defined as the sum of edge lengths along this path. The shortest path length, $L_{ij}$, was defined as the shortest length of the path for nodes i and j; for the network G, the shortest path $L_{p}$ is the average $L_{ij}$ across all the paired nodes. $L_{p}$ quantifies the ability for information propagation, with larger $L_{p}$ indicating lower propagation efficiency.

Normalized Shortest path was defined as $\lambda=\frac{L_{p}}{L_{random}}$, and normalized clustering coefficient was defined as γ=𝐶𝑝𝐶$\gamma=\frac{C_{p}}{C_{random}}$.

The small-worldness was defined as $\sigma=\frac{\gamma}{\lambda}$

Nodal property

Betweenness centrality:

Betweenness centrality of node i:

$$b_{i}=\frac{1}{(n-1)(n-2)}\sum_{\begin{aligned} h,j\in N \\ h\neq j,h\neq i,j\neq i \end{aligned}} \frac{\rho_{hj}(i)}{\rho_{hj}}$$

Where $\rho_{hj}$ is the number of shortest paths between h and j, and $\rho_{hj}(i)$ is the nuber of shortest paths between h and j that pass through i.

Betweenness centrality, defined as the fraction of all shortest paths in the network that pass through a given node. Bridging nodes that connect disparate parts of the network often have a high betweenness centrality. The notion of betweenness centrality is naturally extended to links and could therefore also be used to detect important anatomical or functional connections. The calculation of betweenness centrality has been made significantly more efficient with the recent development of faster algorithms.

Strength:

Strength of node i:

$$S_{i}=sum\left( w_{ij} \right), j=1,2,3,\ldots,N$$

Strength, is one of the most common measures of centrality. The degree has a straightforward neurobiological interpretation, nodes with a high strength are interacting, structurally or functionally, with many other nodes in the network.

Efficiency:

Efficiency of node i:

$$E_{i}=\frac{1}{n}\sum_{i\in N} \frac{\sum_{j\in N,j\neq i} \left( d_{ij}^{w} \right)^{-1}}{n-1}$$

Global efficiency of whole network was defined as:

$$E_{\mathrm{global}}=\frac{1}{N}E_{i}$$

Local Efficiency of whole network was defined as follow:

$$E_{\mathrm{local}}=\frac{1}{n}\sum_{i\in N} E_{\mathrm{global}}(G_{i})$$

Where Gi denotes the subgraph composed of the nearest neighbors of node i

Reference from (Rubinov and Sporns 2010)

Reference

Rubinov, M., and Sporns, O. (2010). "Complex network measures of brain connectivity: uses and interpretations." Neuroimage, 52(3), 1059-69.
